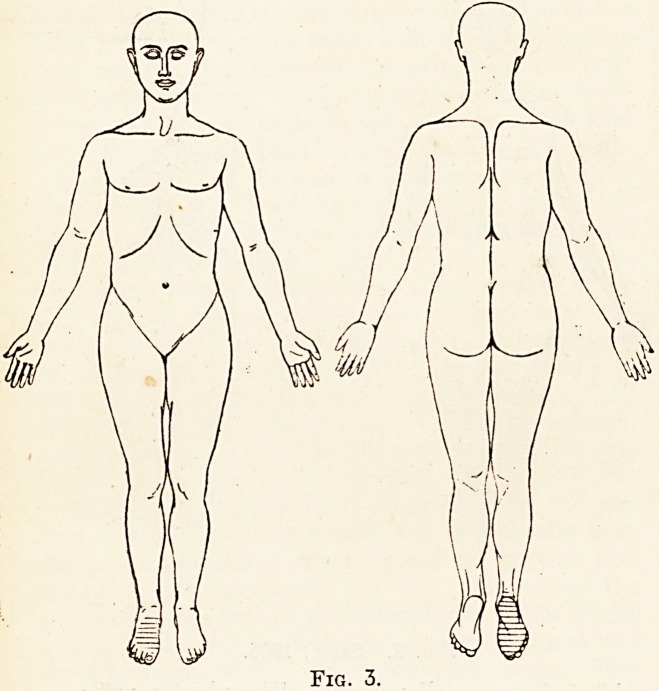# "Rudimentary" Forms of Tabes

**Published:** 1907-12-14

**Authors:** Purves Stewart


### Notes on Current Neurology.

### "RUDIMENTARY" FORMS OF TABES.

By PURVES STEWART, M.D., F.R.C.P.

By way of illustrating this condition, let me recount a couple of examples:—Five and a half years ago a business man, aged 46, consulted me, complaining of a feeling of constriction across the front of the chest in its lower part, chiefly on raising the arms above the head or on stooping forwards. This symptom came on after an attack of influenza two years previously. He also had paroxysms of "red-hot" pain in the fronts of the legs and thighs, lasting for hours at a time, without apparent cause. And he had exquisite tenderness of the skin of the back, so that it was agony for him to dress or undress. At the age of 31 he had gonorrhœa, followed a few weeks later by a slight sore patch on one leg, but he had no hard sore, no throat symptoms, and no cutaneous eruption. A year prior to the influenza he had an accidental fall, in which he cut his face and bruised his chest. He was of temperate habits, married, and had three healthy children.

On examination the pupils were equal and circular, but did not react to light, though contracting briskly on convergence; the cranial nerves were otherwise normal. He had no motor weakness or ataxy of upper or lower limbs. There was intense cutaneous hyperæsthesia to light touches on the abdomen, lower part of chest and slightly on the upper limbs in the area indicated in the accompanying diagram. This accounted for his pain on dressing and undressing.

Fig. 1.—March, 1902.
Cutaneous hyperæsthesia = crosses.
Cutaneous analgesia = horizontal shading.

There was analgesia to pin-pricks on the lower limbs and trunk as high as the second ribs, and along the ulnar borders of the upper limbs, including the two ulnar fingers of each hand. His subjective feeling of constriction, on the front of the chest only, extended from the upper border of the hyperæsthetic area up to the second ribs. Joint-sense was everywhere normal. The elbow-jerks, knee-jerks, and

Fig. 2.—Sept., 1903.

ankle-jerks were all present. The plantar reflexes were brisk and of normal flexor type; the epigastric and abdominal reflexes were increased. The sphincters were normal. The heart, lungs, abdominal organs and urine were all normal. When seen a year and a half later the pains in the legs and constriction feeling across the chest were still present, though less severe, and the hyperæsthesia of the trunk on dressing was less acute. The area of analgesia had markedly diminished in extent, as seen in the accompanying charts (fig. 2), and only included the inner borders of the upper limbs, and part of the lower limbs from the areas of the third lumbar to the first sacral roots inclusive. The cutaneous hyperæsthesia was also less extensive. There was very slight ataxia, the patient walking, as he said (he was a Scotchman), with a "heather-step." The left ankle-jerk was diminished, the left knee-jerk was now absent, the bladder was unaffected. The left side of the palate was slightly weak, its raphé deviating to the right on phonation.

The second case is that of a widow, aged 55, who came to my out-patients last month at the West-End Hospital for Nervous Diseases, complaining of pain and numbness in the left foot of four years' duration. The pain has been so severe as to require morphia injections at several other hospitals where she had been an in-patient. There is no definite history pointing to specific disease. She has one daughter, 24 years of age, and healthy. The cause of death in her husband's case cannot be ascertained. On examination this patient's pupils are equal, and react briskly to light and convergence. The cranial nerves are normal. There is no cutaneous anæsthesia to light touches. There is an area of analgesia on the sole of the right foot and part of the dorsum (see fig. 3). There is loss of vibration-sense in the bones of the left foot only. There is absence of pain on pinching either tendo Achillis, and also on pinching either ulnar nerve above the elbow. There is no

Fig. 3.

weakness or ataxia of upper or lower limbs. The knee-jerks are brisk. The right ankle jerk is present, the left is absent. The cerebro-spinal fluid contains a large excess of lymphocytes, no fewer than 233 per cubic millimetre.

Our conception of tabes dorsalis has become considerably broader within recent years. Ten or twelve years ago the diagnostic signs on which reliance was placed were the Argyll-Robertson pupil, ataxia of gait (hence the name "locomotor ataxia"), and loss of the knee-jerk. But nowadays we are able to recognise the disease by other evidences even in the absence of all these cardinal signs. Thus only in the first of these two cases was the Argyll-Robertson phenomenon present in the pupils, in neither was there any ataxia when the patient came under observation, and in both cases the knee-jerks were present. The classical text-book descriptions of tabes are drawn, and, rightly so, from well-marked cases. But it is important to recognise that some cases take many years before they become "typical"; in fact, some of them never reach the stage of ataxia or loss of knee-jerks. On what signs, then, are we to base our diagnosis? In the first of these cases the original diagnosis was founded on the characteristic sensory changes, which space prevents me from discussing here, together with the presence of the Argyll-Robertson pupillary phenomenon. In the second case the unilateral loss of ankle-jerk (whose significance is similar to that of the knee-jerk), the analgesia of the ulnar nerve-trunks and Achillis tendons, and the sensory changes in the affected foot were, when taken together, suspicious. The condition, however, might have been mistaken for an obscure neuritis. In fact, it had been treated as such by several physicians. But the marked lymphocytosis of the cerebro-spinal fluid placed the diagnosis beyond doubt.

The recognition of such "rudimentary" cases is not only of scientific interest, but has an important bearing on treatment, especially with regard to the prevention of ataxia and of bladder trouble, two of the most important symptoms in advanced cases. Ataxia, I believe, can be delayed and possibly prevented, by the avoidance of physical over-exertion in the lower limbs, whilst bladder trouble, as Edinger long ago pointed out, can also be prevented by instructing the patient to empty the bladder every two hours during the day, whether he feels the inclination or not. If we prevent bladder trouble we avoid the chief dangerous complication of tabes, namely cystitis, and its subsequent ascending kidney infection. In the first of these two patients, who has now been under observation for nearly six years, the bladder is still normal, and the disease has made but slight progress in the way of physical signs; in fact, in some respects the sensory phenomena have actually improved.

## Figures and Tables

**Fig. 1. f1:**
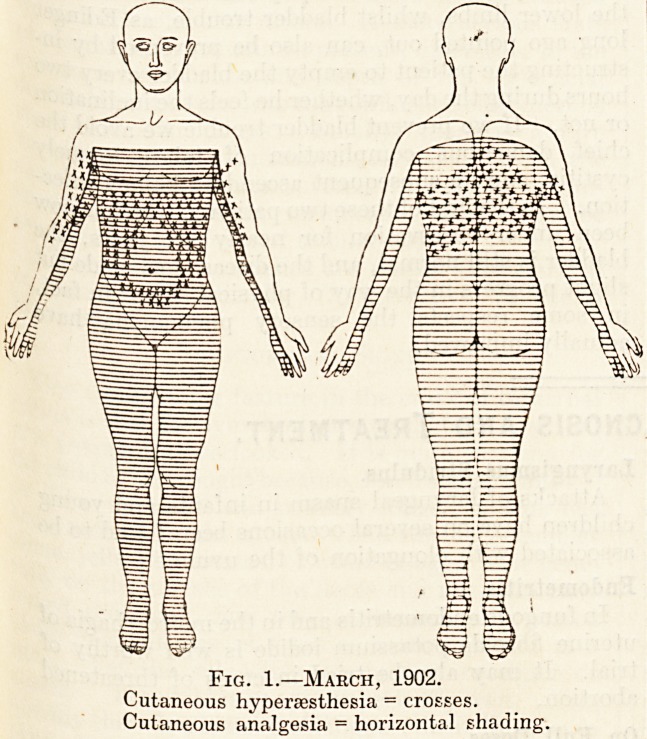


**Fig. 2. f2:**
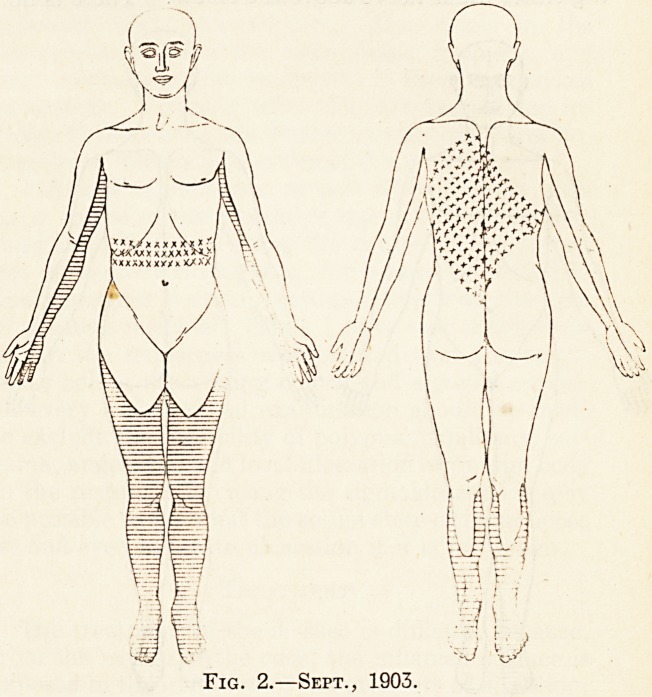


**Fig. 3. f3:**